# Vascular Adventitia Calcification and Its Underlying Mechanism

**DOI:** 10.1371/journal.pone.0132506

**Published:** 2015-07-06

**Authors:** Na Li, Wenli Cheng, Tiequn Huang, Jie Yuan, Xi Wang, Meiyue Song

**Affiliations:** 1 Department of Health Care, China-Japan Friendship Hospital, Ministry of Health, Beijing, China; 2 Center for Cardiovascular Diseases, China-Japan Friendship Hospital, Ministry of Health, Beijing, China; 3 Graduate School, Beijing University of Traditional Chinese Medicine, Beijing, China; University of Iowa, UNITED STATES

## Abstract

Previous research on vascular calcification has mainly focused on the vascular intima and media. However, we show here that vascular calcification may also occur in the adventitia. The purpose of this work is to help elucidate the pathogenic mechanisms underlying vascular calcification. The calcified lesions were examined by Von Kossa staining in ApoE−/− mice which were fed high fat diets (HFD) for 48 weeks and human subjects aged 60 years and older that had died of coronary heart disease, heart failure or acute renal failure. Explant cultured fibroblasts and smooth muscle cells (SMCs)were obtained from rat adventitia and media, respectively. After calcification induction, cells were collected for Alizarin Red S staining. Calcified lesions were observed in the aorta adventitia and coronary artery adventitia of ApoE-/-mice, as well as in the aorta adventitia of human subjects examined. Explant culture of fibroblasts, the primary cell type comprising the adventitia, was successfully induced for calcification after incubation with TGF-β1 (20 ng/ml) + mineralization media for 4 days, and the phenotype conversion vascular adventitia fibroblasts into myofibroblasts was identified. Culture of SMCs, which comprise only a small percentage of all cells in the adventitia, in calcifying medium for 14 days resulted in significant calcification.Vascular calcification can occur in the adventitia. Adventitia calcification may arise from the fibroblasts which were transformed into myofibroblasts or smooth muscle cells.

## Introduction

Previously vascular calcification is considered passive and degenerative. Now it is recognized as a pathobiological process similar to embryonic bone formation. Vascular calcification is a common characteristic associated with atherosclerosis, hypertension, diabetes, vascular lesions, chronic kidney disease, and aging. Increased stiffness and decreased compliance in the vascular wall are the main consequences of vascular calcification, leading to myocardial ischemia, left ventricular hypertrophy, and heart failure, causing thrombosis and plaque rupture, these are part of pathological changes for myocardial ischemia/myocardial infarction. As a result, vascular calcification is thought to be one of the most important factors influencing cardiovascular- and cerebrovascular-related diseases with high morbidity and high mortality rates [1–10].

Mechanical, inflammatory, metabolic, and morphogenetic signals might be involved in vascular calcification. The pathophysiology of vascular calcification is a complex process involving multiple cell types. Anatomically vascular calcification is typically divided into two categories: intimal atherosclerotic calcification and arterial medial calcification [11–12].

Previous research on vascular calcification has mainly focused on the arterial intima and media [1–2,12–13]. However, we show here that vascular calcification may also occur in the adventitia. So far, no one has reported adventitia calcification. In Timothy Ellam’s study, we also find out the evidence of adventitia calcification in ApoE-/- mice (Fig 4c in reference 14) [14]. In Yu-Bin Sui’s study, SD rats treated by intramuscular injections of vitamin D3 and feeding of nicotine also suffered from adventitia calcification (Fig 6Ae in reference 15)[15]. This finding increases the complexity of vascular calcification. Therefore, additional studies are required to elucidate the underlying pathogenic mechanisms governing vascular calcification.

## Materials and Methods

### Animals and treatments

Male Sprague–Dawley (SD) rats (150 ± 10 g) (n = 3) and six-week-old male ApoE-deficient mice (n = 60) were obtained from the Animal Center, Peking University Health Science Center (Beijing). Animals were housed under standard conditions (room temperature 20 ± 8°C, humidity 60 ± 10%, lights from 6:00 to 18:00) and provided standard rodent chow and water freely.At 8 weeks of age, ApoE-deficient mice were switched to a high fat diet (HFD), consisting of 21% fat from lard and 1.25% (wt/wt) cholesterol for an additional 48 weeks.

### Tissue collection and processing

ApoE-deficient mice were sacrificed after anesthetizing with sodium barbital. Hearts and aortas were harvested under sterile conditions. Aortas were fixed in 10% formalin for histological and immunohistochemical observation. Aortic slices were collected consecutively from the beginning of the aortic valve to the point where the aortic valve disappeared (50 μm length and 5 μm slice thickness). Aortas were also evaluated in a 3 mm section on the right side beneath the opening of the innominate artery. Bodies were stored at 4°C, and autopsy was performed by the attending pathologist.

### Human samples

Between December 1984 and January 2010, a total of 20 patients (14 males, 6 females) who were older than 60 years of age and who had died of coronary heart disease, heart failure (left ventricular ejection fraction<45%) or acute renal failure were enrolled in the study. They all had a history of coronary heart disease for more than 10 years, mean age 71.2 ± 5.9. Patients with chronic renal disease or hyperparathyroidism were excluded from the study. Patients were referred to the Department of Pathology of the China-Japan Friendship Hospital for autopsy. All procedures met the appropriate criteria for the use and handling of human tissue in China.

### Consent statement

The study was approved by the Ethics committee of the China-Japan Friendship Hospital(No.2015-37) and was in accordance with the Helsinki Declaration. Written informed consents were obtained from all participants. All animals received humane care in compliance with the Animal Management Rule of the Ministry of Health, People’s Republic of China (documentation no. 55, 2001) and the Care and Use of Laboratory Animals published by the US National Institutes of Health (NIH Publication No. 85–23, revised 1996).

### Materials

Primary antibodies against vimentin, and all secondary antibodies, were purchased from Abcam (Cambridge, UK). Primary antibodies against smooth muscle (SM) α-actin and β-Glycerophosphate and Alizarin Red S were from Sigma (St. Louis, MO, USA). Recombinant transforming growth factor-1 (TGF-β1) was purchased from Pepro Tech Inc (Rocky Hill, NJ, USA). All chemicals and reagents were of analytical grade.

### Vascular smooth muscle cell (VSMC) culture *in vitro*

Explant culture of VSMCs has been previously described [16]. Briefly, thoracic aortas from male SD rats were cut into small pieces after the removal of endothelium and adventitia. These sections were then placed in Dulbecco’s modified Eagle’s medium (DMEM) containing 20% fetal bovine serum (FBS) and maintained at 37°C in an incubator containing 95% air and 5%CO2. VSMCs migrating from explants were collected and maintained in growth medium (DMEM containing 10%FBS). Detection of α-actin in cultured cells confirmed a positive response. VSMCs at passages five to eight were used for experiments.

### Fibroblast culture *in vitro*

Explant culture of fibroblasts has been previously described [17]. Thoracic aorta adventitia from male SD rats were cut into small pieces after the removal of endothelium and media. These sections were placed in DMEM containing 20% FBS and maintained at 37°C in an incubator containing 95% air and 5%CO2. Fibroblasts migrating from explants were collected and maintained in growth medium (DMEM containing 10%FBS). Detection of vimentin in cultured cells confirmed a positive response. The morphology and growth characteristics of the cells were typical of fibroblasts and were distinguished from smooth muscle cells by the absence of ‘hill-and-valley’ growth pattern and the lack of α-actin staining at passages one. Fibroblasts at passages five to eight were used for experiments.

### Calcification of cells *in vitro*

To induce calcification, confluent VSMCs were treated with 2.5 mmol/L CaCl_2_ and 5 mmol/L β-glycerophosphate for 14 days.

To induce calcification, confluent fibroblasts were randomly divided into control group(DMEM containing 1% FBS), mineralization group (DMEM containing 1% FBS, 50 ug/ml ascorbic acid, 5 mmol/L β-glycerophosphate), TGF-β1 (20 ng/ml) + mineralization group (DMEM containing 1% FBS, 50 ug/ml ascorbic acid, 5 mmol/L β-glycerophosphate). The cells (n = 3 wells/group) were treated for 4 days.

After calcification induction, cells were collected for Alizarin Red S staining or the detection of α-actin. Each experiment was performed at least three times in triplicate.

### Alizarin Red S staining

Alizarin Red S staining was performed as previously described with minor modification [16]. Cultured cells grown in 12-well plates were fixed in 4% formaldehyde in phosphate-buffered saline (PBS) for 45 min at 4°C. Samples were, then washed in distilled water and exposed to Alizarin Red S (2% aqueous, Sigma) for 5 min. They were then washed again with distilled water and observed by microscopy. Positive staining is represented as a red/purple color.

### Histological and immunohistochemical staining

Paraffin sections were deparaffinized in xylene, dehydrated indecreasing alcohol solutions, and stained with Von Kossa for calcium deposition. For the detection of α-actin (A2547, Sigma, USA), frozen slices of aorta were fixed in acetone and incubated with monoclonal antibody against α-actin at a dilution of 1:200 at room temperature. For the detection of Vimentin (ab92547, Abcam, UK), fibroblasts were incubated with monoclonal antibody against Vimentin at a dilution of 1:100. Cultured cells were incubated with monoclonal antibody against actin at a dilution of 1:200.

After washing, the slices were incubated in the dark with FITC-labeled secondary antibody at room temperature. Samples were then observed under a fluorescence microscope (Nikon 90i, Japan).

### Statistical analysis

Statistical analysis involved use of SPSS v15(SPSS Inc., Chicago, IL, USA). Data are expressed as means ± SME. Comparisons between two groups involvedt test. A twotailedP< 0.05 was considered statistically significant.

## Results and Discussion

### Calcification in the aorta adventitia of ApoE-/-mice

**Fig 1:**
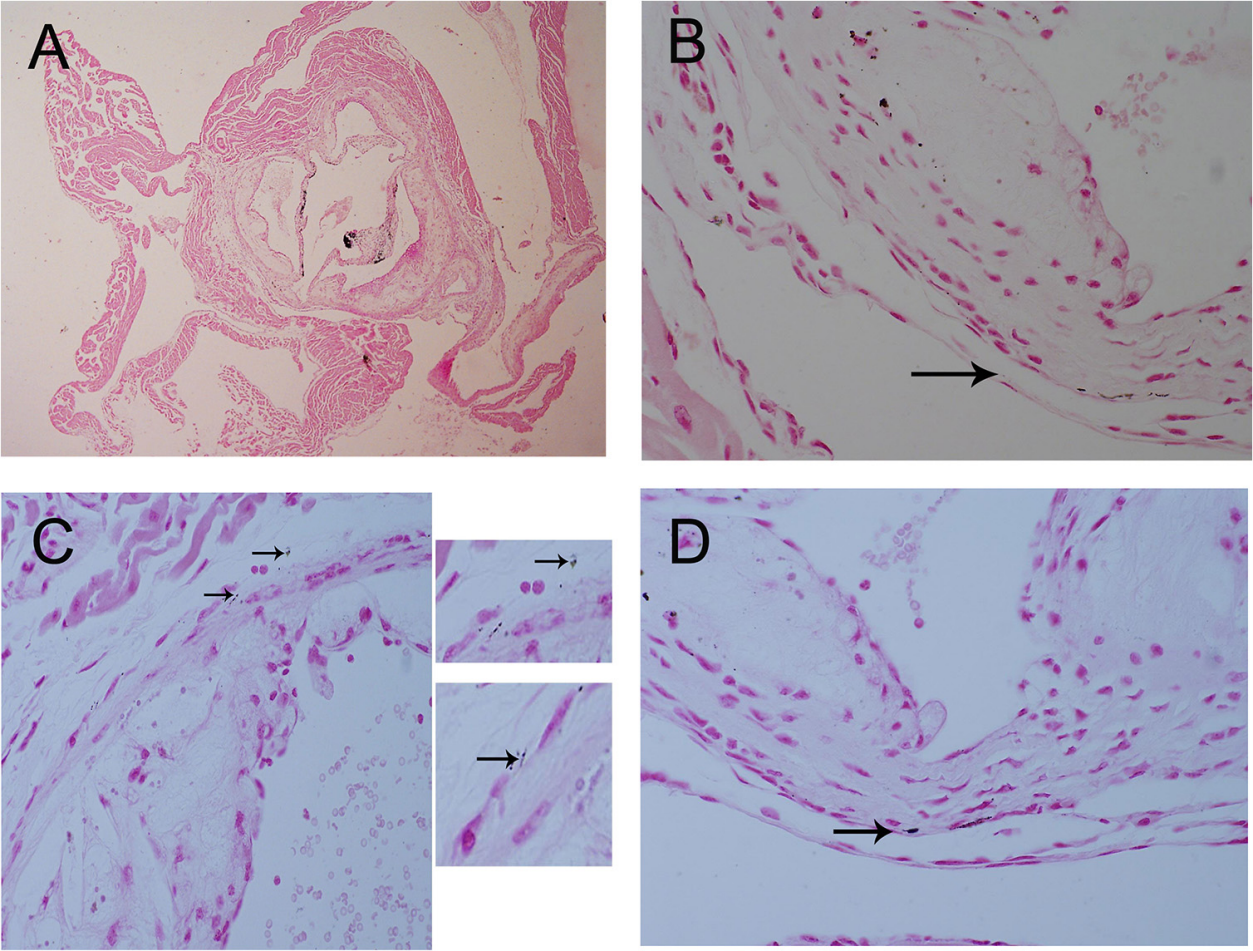
Calcification in the 56-week-old ApoE-/-mice aorta artery by Von Kossa staining. Von Kossa staining showing calcification in the 56-week-old ApoE-/-mice aorta adventitia, intima, and media. Magnification*40 (Fig 1A), *600 (Fig 1B–1D). Arrows indicate calcification in adventitia.

**Fig 2:**
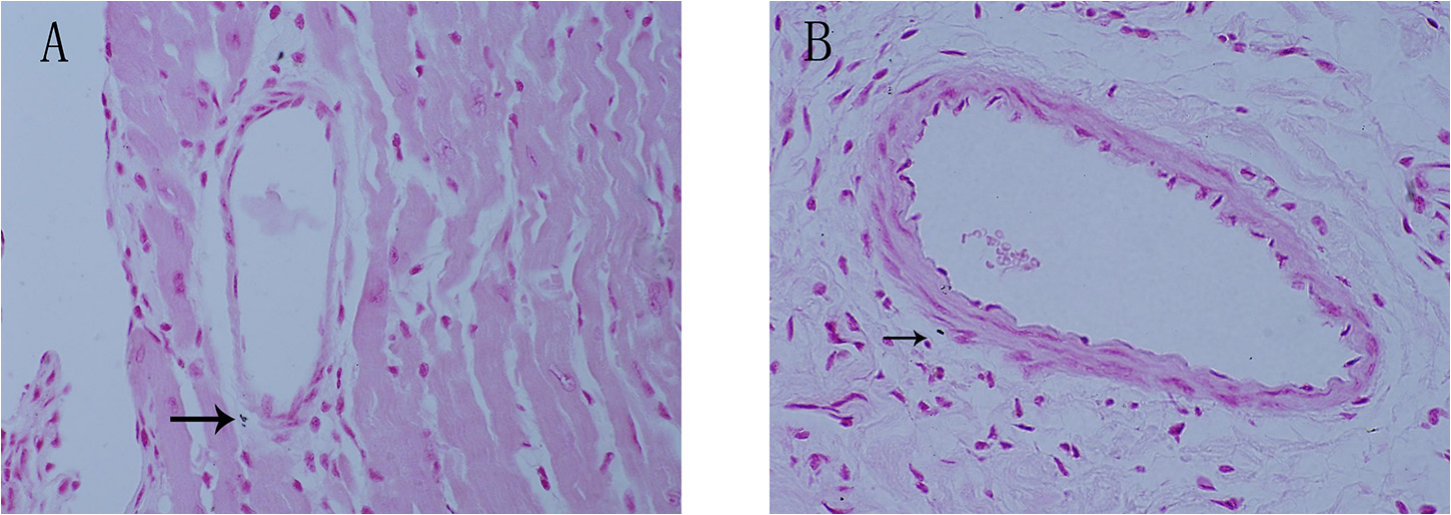
Calcification in the 56-week-old ApoE-/-mice coronary artery. Von Kossa staining showing calcification in the 56-week-old ApoE-/-mice coronary artery, adventitia, and intima. Magnification *600 (Fig 2A.2B). Arrows indicate calcification in adventitia.

After 48 weeks of being fed a HFD, significant calcified lesions were observed in the aorta adventitia of ApoE-/-mice. Calcified lesions were also detectable in the intima and media (Fig 1) and in the coronary artery adventitia and intima (Fig 2). Calcified areas were detectable by Von Kossa staining. Adventitia calcification was observed in 10 percent ApoE-/-mice samples.

### Calcification in the human aorta adventitia

**Fig 3:**
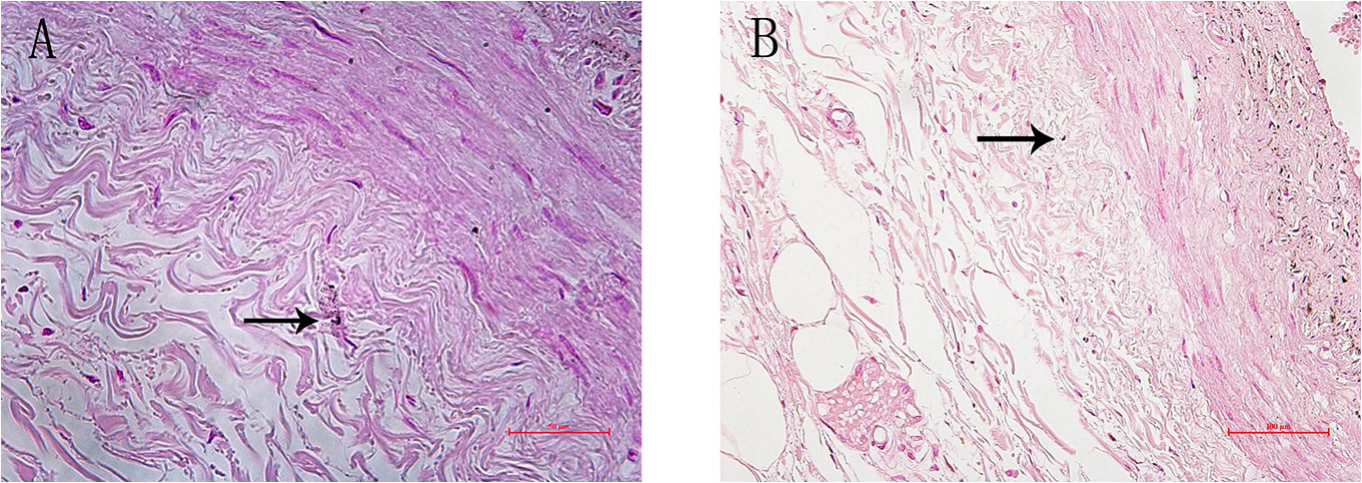
Calcification in the human thoracicaorta adventitia. Von Kossa staining showing calcification in the human **thoracic**aorta adventitia. The human were older than 60 years of age and had died of coronary heart disease, heart failure or acute renal failure. Arrows indicate calcification in adventitia.

Adventitia calcification was observed in 50 percent human samples (Fig 3). The human were older than 60 years of age and had died of coronary heart disease, heart failure or acute renal failure. They all had a history of coronary heart disease for more than 10 years.

### VSMC calcification *in vitro*

**Fig 4:**
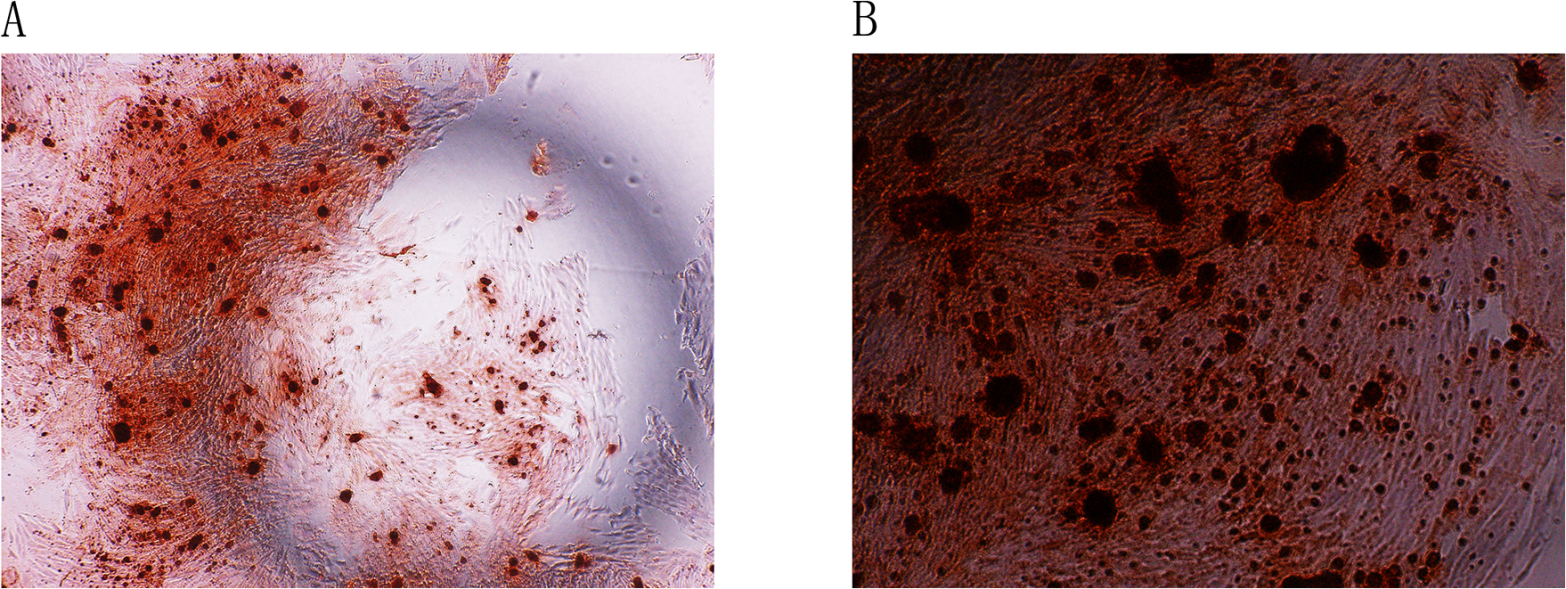
Alizarin Red S staining for VSMCs. Alizarin Red S staining for VSMCs on day 14. Magnification *40 (Fig 4A), *100 (Fig 4B).

To reproduce the same effect *in vitro*, we established primary VSMCs in culture. After incubation with calcifying media for 14 days, significant calcification was detected in VSMCs by Alizarin Red S staining (Fig 4).

### Myofibroblasts calcification *in vitro*

**Fig 5:**
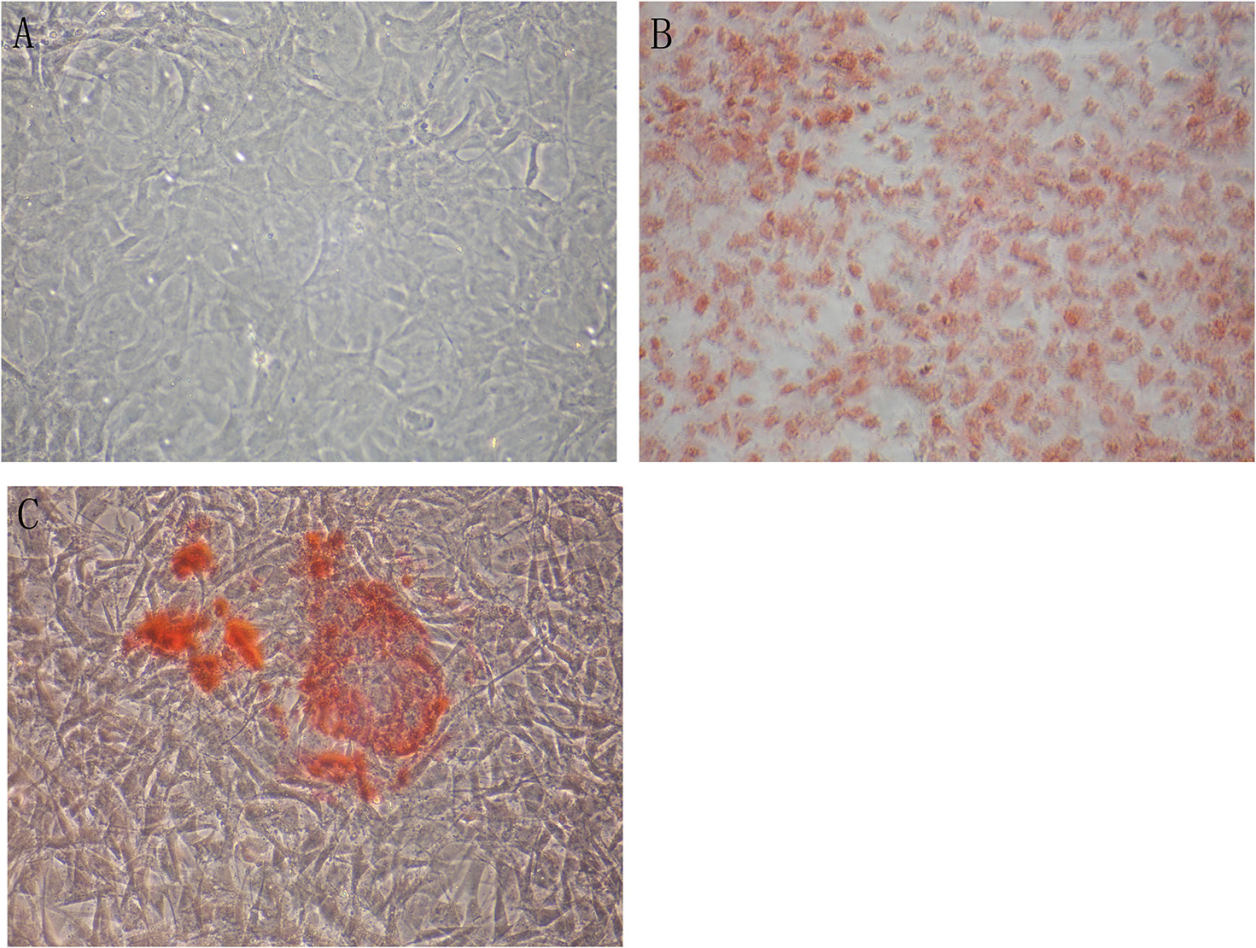
Alizarin Red S staining was positive for myofibroblasts. Fibroblasts were incubated with control media for 4 days. Alizarin Red S staining of these cells was negative(Fig 5A). Calcification was not induced in the necrotic fibroblasts of mineralization group after incubation with mineralization media (DMEM containing 1% FBS, 50 ug/ml ascorbic acid, 5 mmol/L β-glycerophosphate) for 4 days. False positive was detected in fibroblasts by Alizarin Red S staining (Fig 5B).Calcification was detected in myofibroblasts of TGF-β1 (20 ng/ml) + mineralization group by Alizarin Red S staining (Fig 5C). Expression of α-actin in the myofibroblasts. Control group (Fig 6A), mineralization group (Fig 6B), TGF-β1 (20 ng/ml) + mineralization group (Fig 6C). Magnification *200

Successful *in vitro* culture of fibroblasts was confirmed by Vimentin staining. Fibroblasts were then incubated with control media for 4 days. Alizarin Red S staining of these cells was negative (Fig 5A). In contrast to VSMCs, calcification was not induced in the necrotic fibroblasts of mineralization group after incubation with mineralization media (DMEM containing 1% FBS, 50 ug/ml ascorbic acid, 5 mmol/L β-glycerophosphate) for 4 days. False positive was detected in fibroblasts by Alizarin Red S staining (Fig 5B). Calcification was detected in myofibroblasts of TGF-β1 (20 ng/ml) + mineralization group by Alizarin Red S staining (Fig 5C).

**Fig 6:**
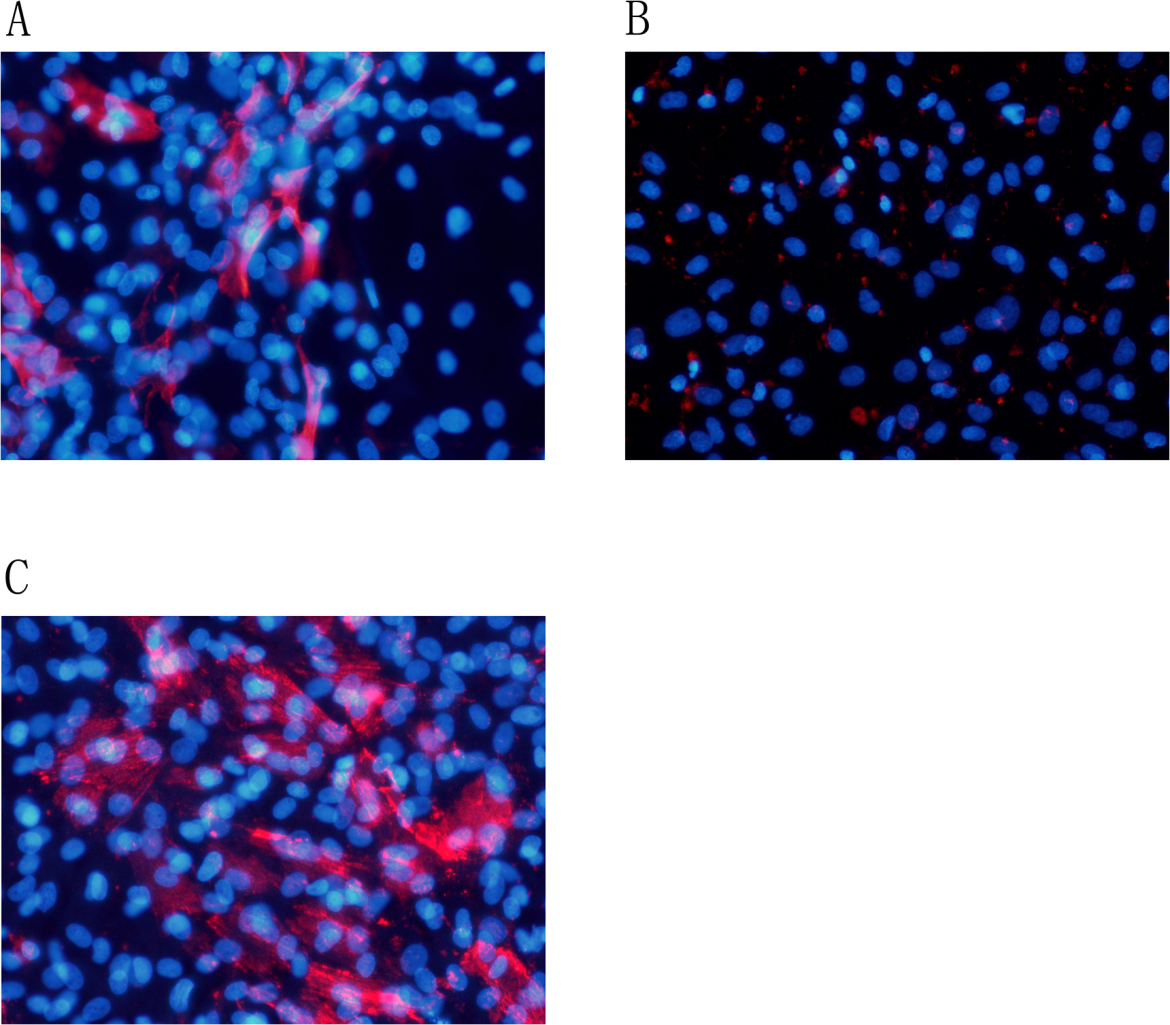
Alizarin Red S staining was positive for myofibroblasts. Fibroblasts were incubated with control media for 4 days. Alizarin Red S staining of these cells was negative(Fig 5A). Calcification was not induced in the necrotic fibroblasts of mineralization group after incubation with mineralization media (DMEM containing 1% FBS, 50 ug/ml ascorbic acid, 5 mmol/L β-glycerophosphate) for 4 days. False positive was detected in fibroblasts by Alizarin Red S staining (Fig 5B).Calcification was detected in myofibroblasts of TGF-β1 (20 ng/ml) + mineralization group by Alizarin Red S staining (Fig 5C). Expression of α-actin in the myofibroblasts. Control group (Fig 6A), mineralization group (Fig 6B), TGF-β1 (20 ng/ml) + mineralization group (Fig 6C). Magnification *200

A-actin was stained positive in 9.3%±1.5% of cells in control group (Fig 6A). False positive was detected in mineralization group (Fig 6B). A-actin positive was observed in 40.4%±4.8%cells of TGF-β1 (20 ng/ml) + mineralization group (DMEM containing 1% FBS, 50ug/ml ascorbic acid, 5 mmol/L β-glycerophosphate)(P<0.01)(Fig 6C). The phenotype conversion of vascular adventitial fibroblasts into myofibroblasts was identified. Quantification of α-actin positive rate was performed by randomly taking five microscopic fields and the mean was calculated.

### A-actin expression

**Fig 7:**
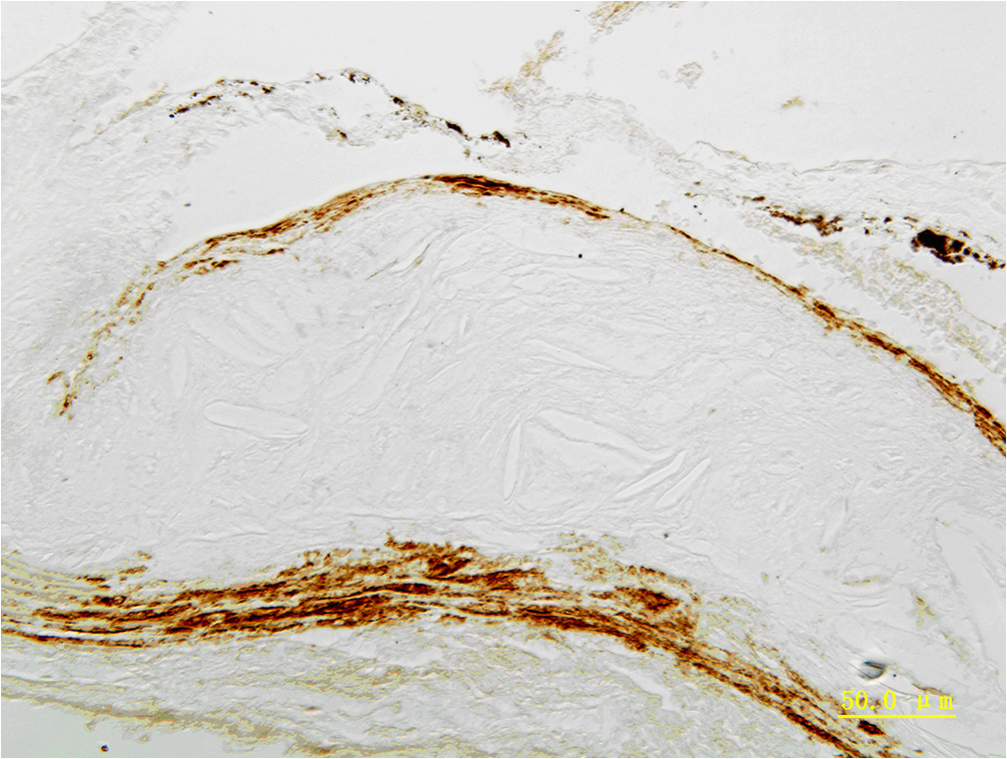
Expression of a-actin in the ApoE-/-mice aorta. Expression of a-actin in the ApoE-/-mice aorta adventitia, media, and intima. Magnification *400.

α-actin expression in the ApoE-/-mice aorta adventitia, media, and intima (Fig 7).

### Adventitia is involved in the formation and progression of atherosclerosis

Traditionally, the primary function of adventitia has been thought to support vessels, provide nutrients for intima, and to maintain sympathetic nerve endings. However, a growing body of evidence indicates that adventitia also actively participates in atherosclerosis, and adventitia inflammation occurs in atherosclerotic disease [18]. Multiple inflammatory cell types, including macrophages, lymphocytes, dendritic cells, and mast cells have been found to infiltrate the adventitia and promote inflammation [19]. Adventitia inflammation in atherosclerotic arteries is widespread and proportional to the severity of atherosclerosis [20]. Importantly, adventitia inflammation can also spread to the intima, promoting development of atherosclerosis in this tissue as well [21].

### Adventitia calcification

In this study, we examined adventitia calcification in atherosclerosis in ApoE-/- mice. Additionally, we found significant levels of calcification in the aorta adventitia of elderly patients.Our results also showed great difference in percentages of adventitia calcification between humans and ApoE-/-mice, which might be associated with the following reasons: 1) in the process that the mouse model of atherosclerosis results in calcification, there is only one single factor, while in the case of human factors are more complex; 2) the relatively smaller human sample size constrained by the experiment condition may be another reason; 3) there may be species difference in adventitia calcification.

However, the mechanisms governing adventitia calcification are not clear, and there have been no previous reports describing a relationship between adventitia inflammation and vascular adventitia calcification. Vascular media and intima calcification result, in part, from proinflammatory Ang II signaling cascades, that involve calpain-1, matrix metalloproteinases -2/9 (MMP-2/9), monocyte chemoattractant protein-1 (MCP-1), TGF-β1 activation and milk fat globule epidermal growth factor-8 (MFG-E8) [22–26]. We propose that adventitia, under the pressure of inflammation and atherosclerosis, undergoes a series of complex changes that contribute to adventitia calcification.

### Cell types involved in adventitia calcification

Adventitia is significantly comprised of collagen fiber and elastic fiber, fibroblast cells, and a small amount of smooth muscle cells. Vascular calcification is associated with several diseases, including hypertension, diabetes, chronic renal failure, and kidney dialysis. In addition, high calcium, high phosphorus, vitamin D3, and nicotine treatment have all been shown to induce vascular calcification in rats. The pathology of vascular calcification involves multiple cell types. For example, smooth muscle cells (SMCs) have been shown to the origin of osteochondroblastic cells [27].

Calcifying vascular cells (CVCs) primarily exist in the vascular wall of the intima. CVCs have characteristics of osteogenic cells, expressing alkaline phosphatase and secreting collagenⅠ, osteopontin (OPN), osteocalcin, and osteonectin. CVCs also display some mesenchymal cell characteristics, which may help promote the trans-differentiation process [28].

Pericytes are star-shaped, multi-functional cells that are found throughout the large, medium, and small artery intima in the inner layer, media outer layer, and the outer nourishing blood vessels. Pericytes can differentiate into macrophages, fat cells, cartilage cells, and smooth muscle cells. Pericytes can also secrete extracellular matrix and form a large number of calcified nodules. They may secrete collagen Ⅰ, OPN, and osteocalcin, which promote differentiation to osteogenic cells, bone cells, and bone tissue [28].

Mesenchymal stem cells, located in vessel walls, also contribute to vascular calcification. This is primarily due to their trans-differentiation to osteogenic cells when exposed to various calcifying factors [29].

Fibroblasts are the major cell type comprising the adventitia, and they are activated in the pathological state. Fibroblasts undergo phenotype transformation, proliferation, and migration. They can also secrete various cytokines, participating in the development of several diseases, including atherosclerosis. Physical damage, hypoxia, and inflammation can cause adventitia fibroblasts to differentiate into myofibroblasts, and this is mainly due to the actions of TGF-1. Myofibroblasts then express smooth muscle actin and collagen. Myofibroblasts contain stress fibers and cytoskeletal proteins with systolic and diastolic function. They can migrate, proliferate, and participate in the formation of the new intima. Adventitia fibroblast migration to the intima is the main cause of new intima thickening and luminal stenosis [30]. In addition, myofibroblasts can secrete a variety of cytokines and growth factors, including TGF-ß1, MCP-1, interleukin (IL), endothelin-1 (ET-1), tumor necrosis factor α (TNF-α), MMPs, and HADPH oxidase, which contribute to adventitia inflammation. For example, TGF-ß1 enhances fibroblast phenotype transformation, and MCP-1 promotes the recruitment and activation of macrophages. ET-1 increases the deposition of extracellular matrix components [31]. Together, these factors promote inflammation. Finally, adventitia fibroblasts, in both physiological and pathological settings, produce reactive oxygen species(ROS), which is involved in oxidative stress.ROS production by adventitia fibroblasts has been shown to be important for cell proliferation and intima regeneration [32–34].

In this study, we show that fibroblasts, the primary cell type comprising the adventitia, succeeded in inducing calcification after incubation with TGF-β1 (20 ng/ml)+mineralization media for 4 days, and identified the phenotype conversion of vascular adventitial fibroblasts into myofibroblasts. Zhang et al. reported the phenotypic differentiation of adventitial fibroblasts into myofibrobalsts from rat aorta [35]. Lai et al. reported that mouse aortic myofibroblasts could induce calcification [17]. Additionally, Simionescu et al. reported that myofibroblasts derived from rat dermal fibroblasts could also induce calcification [36].

Vascular smooth muscle cells (VSMCs) are the primary cell type in the medium, but comprise only a small percentage of cell types found in the adventitia. VSMCs generate and secrete extracellular matrix components and likely contribute to the process of inflammation [37]. VSMCs also have the ability to contract, synthesis, proliferation and secrete multiple factors. The contractile VSMCs, co-exist with pools of proliferative, synthetic, or phagocytic VSMCs, drive the adaptation of vascular function [38]. The contractile VSMCs exists to maintain aortic wall elasticity. Upon mechanical stimulation or molecular signals, transformation of cell phenotype may occur [39]. Transforming the contractile VSMCs into synthetic VSMCs decreases the elasticity of the ascending aorta, and increased collagen deposition hardens the aortic tissue [39].A-SMA/SM22α is a marker of contractile VSMCs in normal, healthy adult tissue. Expression of these markers is significantly decreased in embryonic vascular development or vessel damage repair. OPN is an additional marker that is often used. Upon transforming into synthetic VSMCs, OPN expression increases. In contrast, transforming into contractile VSMCs promotes a decrease in OPN expression.

Here, we show that *in vitro* culture of SMCs with calcification-inducing medium results in strong calcification of these cells. We propose that adventitia calcification also arises from SMCs, which comprise a small percentage of the adventitia cell population. We detected positive expression of a-actin in the aorta adventitia, media, and intima of ApoE-/-mice. These α-actin positive cells in the adventitia could be SMCs but could also be myofibroblasts. The adventitia is mainly comprised of the fibroblasts, as well as tiny amount of smooth muscle cells. Adventitia calcification may arise from the fibroblasts which were transformed into myofibroblasts or smooth muscle cells.

Our subjects only involved the large arteries and did not include the medium and small arteries, which is our limitation of the study.

Recent reports have described other mechanisms that may contribute to the process, including mechanical stimulation, biochemical factors, and micro-RNA regulation. Additional studies are needed to fully understand the process of vascular calcification and cell phenotype transformation. Such studies may help clinical treatment in the future.

## Conclusions

Vascular calcification can occur in the adventitia. The adventitia is mainly comprised of the fibroblasts, as well as tiny amount of smooth muscle cells. Adventitia calcification may arise from the fibroblasts which were transformed into myofibroblasts or smooth muscle cells.
